# Gambogic acid sensitizes gemcitabine efficacy in pancreatic cancer by reducing the expression of ribonucleotide reductase subunit-M2 (RRM2)

**DOI:** 10.1186/s13046-017-0579-0

**Published:** 2017-08-10

**Authors:** Guanggai Xia, Hongcheng Wang, Ziliang Song, Qingcai Meng, Xiuyan Huang, Xinyu Huang

**Affiliations:** 1 Department of general surgery, Shanghai Jiao Tong University Affiliated Sixth People’s Hospital, 600 Yishan Rd, Shanghai, 200233 People’s Republic of China; 2 Department of Pancreatic Surgery, Pancreatic Cancer Institute, Fudan University Shanghai Cancer Center, 270 Dong-An Road, Shanghai, 200032 People’s Republic of China

**Keywords:** Pancreatic cancer, Gemcitabine, Gambogic acid, RRM2

## Abstract

**Background:**

Pancreatic cancer is susceptible to gemcitabine resistance, and patients receive less benefit from gemcitabine chemotherapy. Previous studies report that gambogic acid possesses antineoplastic properties; however, to our knowledge, there have been no specific studies on its effects in pancreatic cancer. Therefore, the purpose of this study was to explore whether increases the sensitivity of pancreatic cancer to gemcitabine, and determine the synergistic effects of gambogic acid and gemcitabine against pancreatic cancer.

**Methods:**

The effects of gambogic acid on cell viability, the cell cycle, and apoptosis were assessed using 4,5-dimethylthiazol-2-yl)-3,5-diphenylformazan (MTT) and flow cytometry in pancreatic cancer cell lines. Protein expression was detected by western blot analysis and mRNA expression was detected using q-PCR. A xenograft tumor model of pancreatic cancer was used to investigate the synergistic effects of gambogic acid and gemcitabine.

**Results:**

Gambogic acid effectively inhibited the growth of pancreatic cancer cell lines by inducing S-phase cell cycle arrest and apoptosis. Synergistic activity of gambogic acid combined with gemcitabine was observed in PANC-1 and BxPC-3 cells based on the results of MTT, colony formation, and apoptosis assays. Western blot results demonstrated that gambogic acid sensitized gemcitabine-induced apoptosis by enhancing the expression of cleaved caspase-3, cleaved caspase-9, cleaved-PARP, and Bax, and reducing the expression of Bcl-2. In particular, gambogic acid reduced the expression of the ribonucleotide reductase subunit-M2 (RRM2) protein and mRNA, a trend that correlated with resistance to gemcitabine through inhibition of the extracellular signal-regulated kinase (ERK)/E2F1 signaling pathway. Treatment with gambogic acid and gemcitabine significantly repressed tumor growth in the xenograft pancreatic cancer model. Immunohistochemistry results demonstrated a downregulation of p-ERK, E2F1, and RRM2 in mice receiving gambogic acid treatment and combination treatment.

**Conclusions:**

These results demonstrate that gambogic acid sensitizes pancreatic cancer cells to gemcitabine in vitro and in vivo by inhibiting the activation of the ERK/E2F1/RRM2 signaling pathway. The results also indicate that gambogic acid treatment combined with gemcitabine might be a promising chemotherapy strategy for pancreatic cancer.

**Electronic supplementary material:**

The online version of this article (doi:10.1186/s13046-017-0579-0) contains supplementary material, which is available to authorized users.

## Background

Pancreatic cancer is highly malignant and has the fourth highest mortality rate among all tumors. Although chemotherapy improves the prognosis in pancreatic cancer patients, the five-year survival rate remains less than 5% [1–3]. Gemcitabine-based chemotherapy is most commonly used in pancreatic cancer [4, 5]. Albumin-bound paclitaxel combined with gemcitabine improves survival in patients with advanced pancreatic cancer; however, those patients had little survival benefit, companied by serious side effects [6, 7]. Therefore, exploration of a novel drug that can be used in combination with gemcitabine is crucial to improving both the therapeutic effects of gemcitabine in pancreatic cancer patients and the prognosis in such patients.

Ribonucleotide reductase (RNR) is an enzyme that regulates the cell cycle, and is composed of two subunits, the regulatory subunit RRM1 and catalytic subunit RRM2 [8–10]. Previous studies have found that the expression of RRM1 and RRM2 in pancreatic cancer cells increases their sensitivity to gemcitabine [11–13]. In addition, clinical studies have shown that high levels of RRM1 or RRM2 are associated with a poor prognosis in both pancreatic and lung cancers [14–16]. However, RRM1 also acts as a tumor suppressor gene that inhibits tumor cell growth and metastasis by inducing the expression of the tumor suppressor gene *PTEN* in lung cancer [17].

In recent years, an increasing number of studies have been conducted on herbal and plant-derived drugs. Evidently, many Chinese herbal medicines can effectively treat cancer patients, improve patient outcomes, and reduce the side effects of chemotherapy drugs [18–20]. Gamboge is a dry resin that is secreted by the *Garcinia hanburyi* tree. Rattan gamboge has anti-tumor effects, and gambogic acid (GA) is one of the main components of gamboge [21]. In vitro *and* in vivo studies have reported that GA inhibits the growth of various tumors, such as those of prostate, lung, stomach, and liver cancer among others [21–24]. Gambogic acid induces apoptosis of tumor cells and destroys cancer cells by increasing the levels of active oxygen, inhibiting the NF-κB, MAPK/ERK, and PI3K/AKT signaling pathways [21]. However, there are few studies on the effects of GA in pancreatic cancer, and the specific mechanisms underlying those effects remain unclear [25, 26].

Therefore, we studied the effects of GA combined with gemcitabine against pancreatic cancer both in vivo and in vitro. The GA treatment was found to enhance the sensitivity of pancreatic cancer cells to gemcitabine by inhibiting the expression of RRM2. Furthermore, the combination of these two drugs synergistically inhibited tumor growth.

## Methods

### Regents

Gambogic acid (98% purity, Yuanye Biotech, China) was dissolved in dimethyl sulfoxide (DMSO) to 20 mg/mL and stored for subsequent use. Gemcitabine (Sigma-Aldrich, USA) was dissolved in water to 50 mM and stored. The extracellular signal-regulated kinase (ERK) inhibitor ulixertinib, proteasome inhibitor MG-132, and pan-caspase inhibitor Z-VAD-FMK (Selleck, USA) were dissolved in DMSO to 10 mM and stored for subsequent use. All regents were stored at a temperature below − 80 °C. A solution of 4,5-dimethylthiazol-2-yl)-3,5-diphenylformazan (MTT) was dissolved in phosphate buffered saline (PBS) and stored at 20 °C. The AnnexinV/PI apoptosis kit was purchased from Vazyme (China), and a Cell cycle detection kit was purchased from Beyotime (China). Primary antibodies against cleaved PARP, cleaved caspase-3, cleaved caspase-9, Bcl-2, Bax, ERK1/2, phospho-ERK1/2, AKT, and phospho-AKT were purchased from Cell Signaling Technology (USA). Primary antibodies against RRM1, RRM2, and E2F1 were purchased from Affinity Bioscience (USA). An IHC (immunohistochemistry) detection kit was purchased from CWBio (China).

### Cell lines and cell culture

Human pancreatic cancer cell lines BxPC-3, PANC-1, MIA PaCa-2, and SW1990 were obtained from the Type Culture Collection of the Chinese Academy of Sciences (Shanghai). The BxPC-3 and SW1990 cell lines were cultured in Roswell Park Memorial Institute (RPMI) 1640 medium supplemented with 10% FBS (fetal bovine serum). The PANC-1 and MIA PaCa-2 cell lines were cultured in Dulbecco’s modified Eagle medium supplemented with 10% FBS. All cell lines were maintained in an incubator at 37 °C with 5% CO_2_.

### Cell viability assay

The MTT assay was performed to detect cell viability following treatment with GA, gemcitabine alone, or the two combined. Cells (8 × 10^3^) were seeded into 96-well culture plates for overnight incubation at 37 °C and treated with varying concentrations (0.0, 0.5, 1.0, and 2.0 μM) of GA. Following treatment with GA for 12, 24, and 48 h, respectively, the culture medium was discarded. Then, 100 μL of the culture medium was mixed with 20% MTT solution and added into wells of the culture plate and incubated for 4 h at 37 °C. The culture medium was again discarded and 150 μL DMSO solution was added into the wells that were further incubated at 37 °C for 15 min. The culture plates were subjected to shaking for 10 mins, after which the absorbance was measured using a microplate reader at 490 nm. For evaluation of the combined treatment with the two drugs, cells were first exposed to GA for 12 h, and then treated with gemcitabine for another 48 h, followed by washout of GA. The combination index (CI) was calculated using the Chou–Talalay method and the CalcuSyn software (Biosoft, UK). A CI value <0.90 indicates synergism; a CI value between 0.90 and 1.10 indicates an additive effect; and a CI > 1.10 indicates antagonism.

### Quantitative PCR analysis

Total RNA of the cells was isolated using TRIzol (Takara, Japan) according to the manufacturer’s instructions. A Reverse Transcription kit (Takara, Japan) and 500 ng RNA were used for cDNA synthesis. A quantitative reverse-transcriptase polymerase chain reaction (qRT-PCR) kit (Takara, Japan) and a 7500 HT Fast Real-Time PCR System (Applied Biosystems, USA) were used for PCR. Data were normalized with glyceraldehyde 3-phosphate dehydrogenase (GAPDH) and presented as an expression fold change using the 2^-ΔΔCt^ method. Primers were synthesized by Sangon Biotech (China). The primer sequences were as follows: RRM1; forward 5′-ATGGCTGCTGTGTTCCTCTC-3′, reverse 5′-CGGCTAATCCAATCCAGTTC-3′; RRM2; forward 5′-CCTCCGATGGTTTGTGTACC-3′, reverse 5′-TGGCTCAAGAAACGAGGACT-3′; and E2F1; forward 5′-GGGGAGAAGTCACGCTATGA-3′, reverse 5′-CTTCTGCACCTTCAGCACCT-3′.

### RNA interference

The E2F1 siRNA was purchased from RiboBio (China). A number of BxPC-3 or PANC-1 cells (2 × 10^5^ each) were seeded into six-well plates for overnight incubation at 37 °C. The E2F1 siRNA (50 nM) was transfected into cells using Lipofectamine 2000 (Invitrogen, USA) and optimum culture medium according to routine protocols. The culture medium was refreshed with normal culture medium following incubation for 4 h. After 2 days, the cells were collected and prepared for extraction of total protein and western blot analysis. The si-E2F1 primer sequences were as follows: forward 5′-GCGCAUCUAUGACAUCACCTT-3′ and reverse 5′-GGUGAUGUCAUAGAUGCGCTT-3′.

### Transfection of RRM2 overexpression plasmid

The RRM2 cDNA was synthesized by RT-PCR, and then cloned into the pcDNA3.1 vector. Transfection of RRM2 overexpression plasmid into PANC-1 and BxPC-3 cells was carried out using Lipofectamine 2000 (Invitrogen) according to routine protocols. After 2 days incubation, cells were collected and prepared for extraction of total protein and western blot analysis. The PCR primer sequence was as follows: forward 5′-CTCGAGATGCTCTCCCTCCGTGTCCC-3′ and reverse 5′-GGATCCAAGTCAGCATCCAAGGTAAA-3′.

### Western blotting

Approximately 1 × 10^6^ cells were cultured in 60 mm dishes, after which they were treated with the respective drugs under investigation for 24 h. Total protein was extracted with RIPA (radioimmunoprecipitation assay) buffer (Beyotime, China), and the protein concentration was detected. Then, total proteins were separated by SDS-PAGE (10%–15%), and electrically transferred into polyvinylidene difluoride (PVDF) membranes. The membranes were then blocked with 5% defatted milk for 1 h at room temperature. They were then incubated with primary antibodies overnight at 4 °C. The membrane was further incubated with an appropriate horseradish peroxidase (HRP)-conjugated secondary antibody for 1 h at room temperature. Protein bands were detected by enhanced chemiluminescence (Thermo Fisher Scientific, USA).

### Apoptosis assay

Approximately 4 × 10^5^ cells were seeded into six-well plates for culture overnight, after which they were treated with varying concentrations (0.0, 0.5, 1.0, and 2.0 μM) of GA for 24 h. For the combination treatment, cells were first exposed to GA for 12 h, and subsequently treated with gemcitabine for another 48 h, followed by the washout of GA. Cells were centrifuged and collected. The FITC-Annexin V and PI solutions were used to dye cells, and apoptosis was detected by a Flow cytometer (Beckman, Navios 2 L 8C, USA). Data were analyzed with the FlowJo v10 software (Ashland, OR, USA).

### Cell cycle assay

Approximately 4 × 10^5^ cells were seeded into six-well plates for culture overnight, after which they were treated with varying concentrations (0.0, 0.5, 1.0, and 2.0 μM) of GA for 24 h. Cells were collected, and fixed by 75% ethanol overnight at 4 °C. Cells were washed with PBS, and incubated with RNaseA at 37 °C in a water bath for 40 min. They were then dyed with PI solution for 20 min at room temperature in the dark, and detection was performed using a Flow cytometer (Beckman, Navios 2 L 8C, USA). Cell cycle distribution and the different cell cycle phase data were analyzed by the ModFit software (Verity Software House, USA).

### Xenograft tumor model

Animal experimental procedures were approved by the Ethical Review Committee of The Sixth Affiliated Hospital of Shanghai Jiao Tong University. Six-week-old Balb/c female nude mice were purchased from Shanghai Si Lai Ke Laboratory Animal Co., Ltd. China. The BxPC-3 cells (5 × 10^6^) were subcutaneously inoculated into the right thigh of each mouse. After the tumor reached a size of about 40 mm^3^, mice were divided into four groups, each comprising five mice. The experimental groups included the control group (saline only); GA group (8 mg/kg, once every 3 days, intraperitoneally); gemcitabine (GEM) group (100 mg/kg, once every 3 days, intraperitoneally); and the combination group (first day treatment of 8 mg/kg GA, followed by second day treatment of 100 mg/kg gemcitabine, administered once every 3 days, intraperitoneally). The volume of all solutions injection was 200 μL. Tumor size and mice weight were measured using an electronic vernier caliper and scale, respectively. Tumor volume was calculated with the formula: Volume = 0.5 × (length × width^2^). After 26 days, mice were euthanized, and the tumors were excised, weighed, and prepared for paraffin embedding.

### IHC analysis

Xenograft tumor tissues were embedded in paraffin, and sliced into 4 μm sections in preparation for IHC staining, as previously described. Tumor sections were deparaffinized, rehydrated, and subjected to antigen-retrieval with citrate buffer at 95 °C for 10 min. The sections were then blocked by goat serum, and incubated with relevant primary antibodies overnight at 4 °C. An appropriate HRP-conjugated second antibody was used to incubate tissue sections, and the targeted antigen was detected with diaminobenzidine (DAB) solution. The IHC evaluation was based on the staining intensity and stained proportion, and five random fields at a 400 × magnification were evaluated. The quantification of IHC was calculated as outlined in a previous study [10]. The score of staining intensity was based on a scale from 0 to 3 points (0, absent; 1, weak; 2, moderate; and 3, intense). The stained proportion was also on a scale of 1–3 points (1, < 10%; 2, 10%–49%; 3, > 50% of cells positive), and the IHC score was calculated by multiplying the two scores.

### Statistical analysis

The SPSS 18.0 software was used for statistical analysis. Data were presented as mean ± SD, and the Student’s *t*-test was used to calculate *P*-value. Two-sided *P*-values <0.05 were considered statistically significant.

## Results

### Gambogic acid effectively inhibits the growth of pancreatic cancer cells

**Fig. 1:**
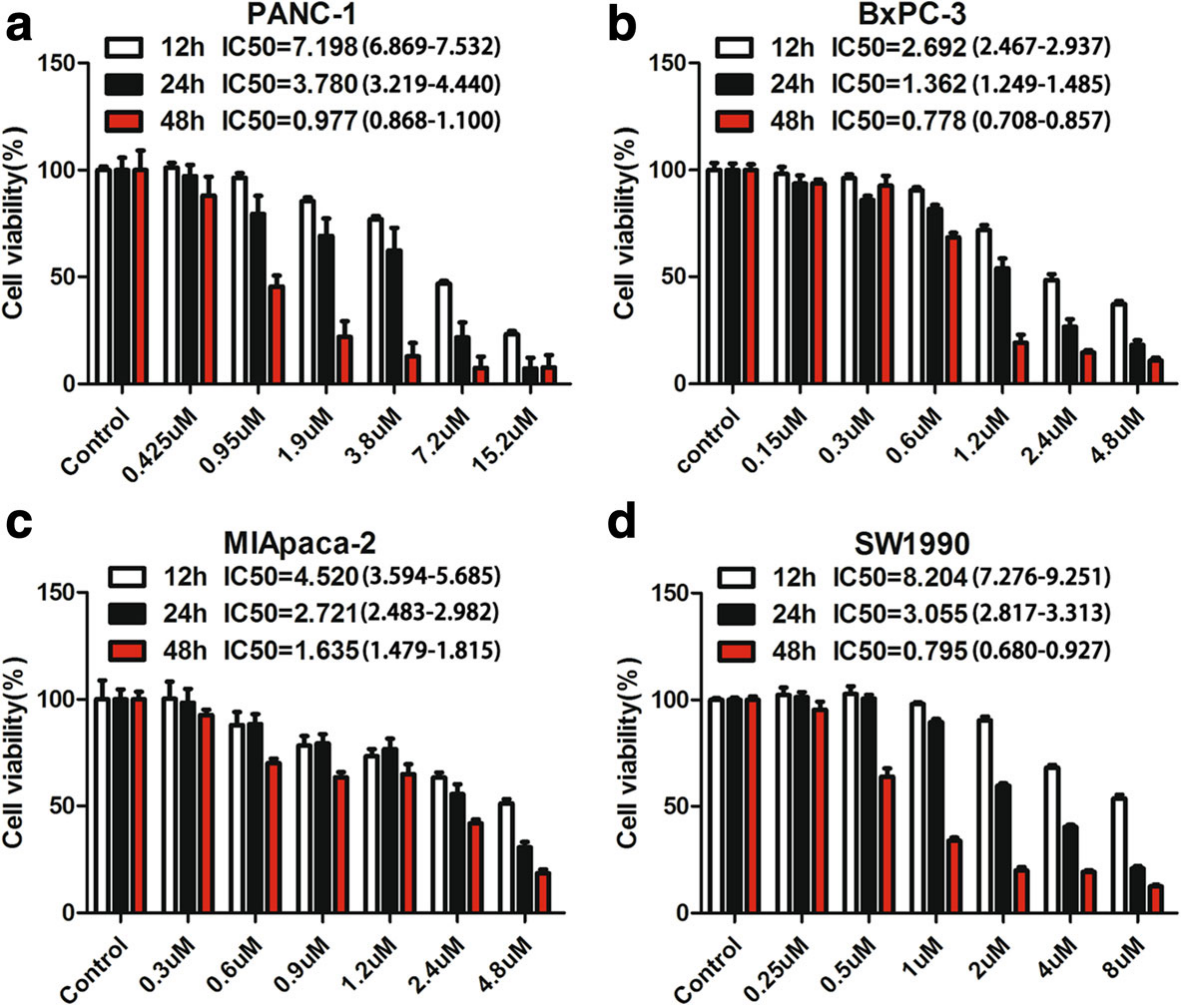
Gambogic acid (GA) inhibited the growth of pancreatic cancer cells. Pancreatic cancer cells were treated with GA at different concentrations for 12, 24, and 48 h, respectively. Cell viability was detected using the 4,5-dimethylthiazol-2-yl)-3,5-diphenylformazan (MTT) assay, and the half maximal inhibitory concentration (IC_50_) was calculated. **a** PANC-1 cells; (**b**) BxPC-3 cells; (**c**) MIA PaCa-2 cells; (**d**) SW1990 cells. The IC_50_ of GA against these cells was calculated. Data are presented as mean ± SD (*n* = 5)

**Fig. 2:**
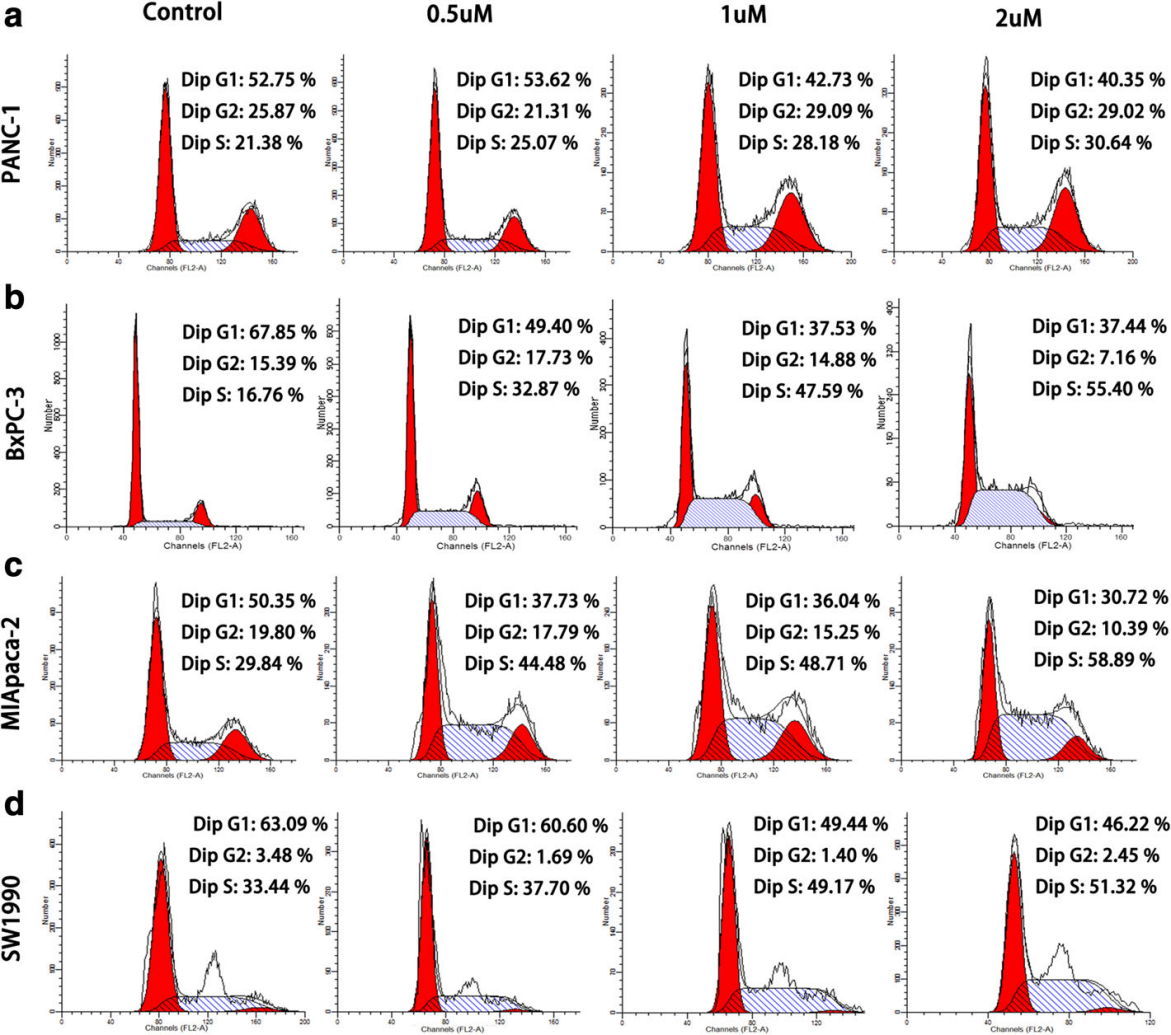
Gambogic acid (GA) induced pancreatic cancer S-phase cell cycle arrest. Pancreatic cancer cells were treated with 0.0, 0.5, 1.0, and 2.0 μM GA for 24 h, respectively, and the cell cycle was evaluated using flow cytometry. **a** PANC-1 cells; (**b**) BxPC-3 cells; (**c**) MIA PaCa-2 cells; (**d**) SW1990 cells

**Fig. 3:**
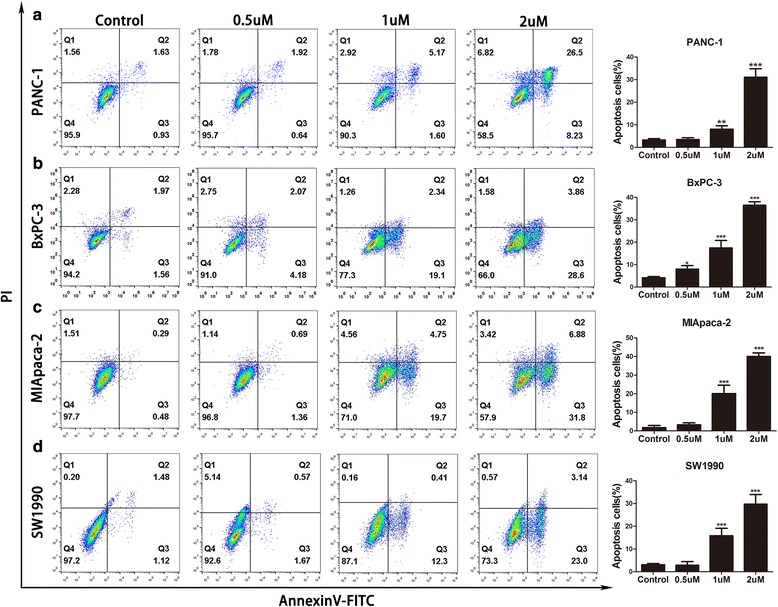
Gambogic acid (GA)-induced apoptosis of pancreatic cancer cells. Pancreatic cancer cells were treated with 0.0, 0.5, 1.0, and 2.0 μM GA for 24 h, respectively, and apoptosis was detected using flow cytometry. **a** PANC-1 cells; (**b**) BxPC-3 cells; (**c**) MIA PaCa-2 cells; (**d**) SW1990 cells. Data are presented as mean ± SD (*n* = 3); ** indicates P<0.01; *** indicates P<0.001

As shown in Fig. 1, the cytotoxicity of GA against pancreatic cancer cells was dose- and time-dependent. The viability of PANC-1, BxPC-3, MIA PaCa-2, and SW1990 cells decreased, with increasing GA concentration and treatment time. In contrast, GA only caused minimal loss of cell viability in human pancreatic ductal epithelial cells (HPDE) (Additional file 1: Figure S1). The half maximal inhibitory concentrations (IC_50_) of these four pancreatic cancer cell lines are shown in Fig. 1. To determine whether the cytotoxicity of GA contributed to its ability to induce apoptosis and affect the cell cycle, we performed apoptosis and cell cycle assays. Cell cycle distribution in the S-phase was observed to be augmented as GA concentration increased (Fig. 2), indicating that GA could induce S-phase arrest in pancreatic cancer cells. Figure 3 shows that GA could induce apoptosis in these four cell lines in a dose-dependent manner. The pan-caspase inhibitor Z-VAD-FMK reduced GA-induced apoptosis in pancreatic cancer cells. This suggests that GA induced apoptosis in pancreatic cancer cells by activating the caspase pathway (Additional file 2: Figure S2). These results demonstrate that GA effectively induces cell death of pancreatic cancer cells.

### Gambogic acid reduces the expression of RRM2 by inhibiting the ERK signaling pathway

**Fig. 4:**
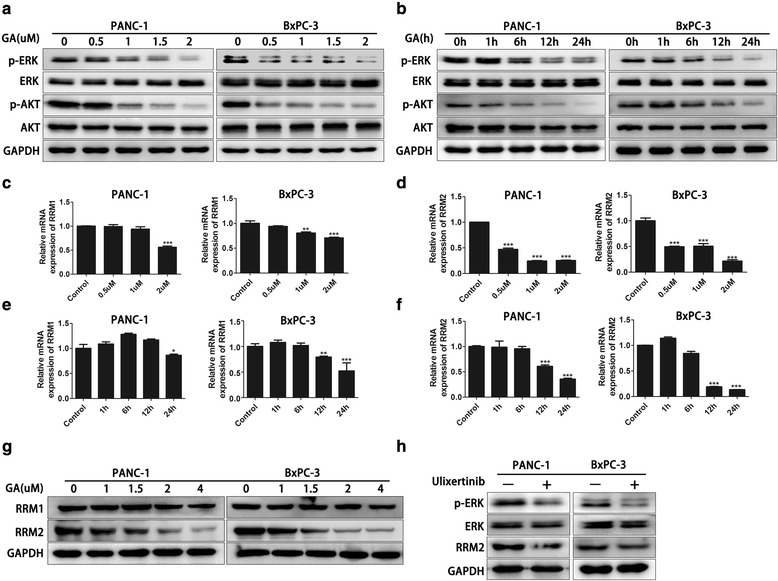
Gambogic acid (GA) reduced ribonucleotide reductase subunit-M2 (RRM2) expression through inhibition of the ERK signaling pathway. **a** PANC-1 and BxPC-3 cells were treated with 0.0, 0.5, 1.0, 1.5, and 2.0 μM, respectively, for 24 h. The protein levels of p-ERK, ERK, p-AKT, and AKT were detected using western blot analysis. **b** PANC-1 and BxPC-3 cells were treated with 2 μM GA for 0, 1, 6, 12, and 24 h, respectively. The protein levels of p-ERK, ERK, p-AKT, and AKT were detected using western blot analysis. **c**, **d** PANC-1 and BxPC-3 cells were treated with 0, 0.5, 1, 1.5, and 2 μM, respectively, for 24 h. The mRNA levels of RRM1 and RRM2 were detected by q-PCR. **e**, **f** PANC-1 and BxPC-3 cells were treated with 2 μm GA for 0, 1, 6, 12, and 24 h, respectively. The mRNA levels of RRM1 and RRM2 were detected by q-PCR. **g** PANC-1 and BxPC-3 cells were treated with 0, 1, 1.5, 2, and 4 μM GA for 24 h, respectively. The protein levels of RRM1 and RRM2 were detected using western blot analysis. **h** PANC-1 and BxPC-3 cells were treated with 1 nM of the ERK inhibitor ulixertinib for 24 h. The protein levels of p-ERK, ERK, and RRM2 were detected using western blot analysis. Data are presented as mean ± SD (*n* = 3); * indicates *P* < 0.05; ** indicates *P* < 0.01; *** indicates *P* < 0.001

The ERK signaling pathway is associated with the cell cycle and apoptosis. A previous study has shown that activation of the ERK signaling pathway could increase cell growth by promoting the cell cycle and inhibiting apoptosis [27]. Thus, we performed western blot analysis to investigate whether GA suppresses the ERK signaling pathway in pancreatic cancer cells. The PANC-1 and BxPC-3 cells were treated with various concentrations of GA and different incubation times. We observed that GA inhibited the phosphorylation of ERK both when the concentration of GA reached 1 μM or above, and the treatment time reached 12 or 24 h (Fig. 4a, b). Inhibition of the ERK signaling pathway reportedly activates the AKT signaling pathway that could also promote cancer cell growth and drug resistance [28, 29]. Thus, we investigated whether GA affects the AKT signaling pathway when ERK signaling pathway is inhibited. The results showed that phosphorylation of AKT could be inhibited by GA (Fig. 4a, b).

As regulators of the cell cycle, RRM1 and RRM2 are associated with gemcitabine resistance in pancreatic cancer. We explored whether GA affects the expression of RRM1 and RRM2 in pancreatic cancer cells. At the mRNA level, the expression of RRM1 declined slightly as the concentration of GA and treatment time increased (Fig. 4c, d). However, RRM2 was significantly suppressed by 2 μM GA after 12 and 24 h treatment, respectively. As the concentration of GA increased, the mRNA fold change was also increased (Fig. 4e, f). At the protein level, we found that RRM2 expression could be inhibited by GA in PANC-1 and BxPC-3 cells. However, RRM1 expression was only slightly affected by treatment with 4 μM GA (Fig. 4g), indicating that GA could more effectively inhibit the expression of RRM2 compared to that of RRM1. Therefore, our study subsequently focused only on RRM2. We also found that levels of the RRM2 protein could be inhibited by the ERK inhibitor (Fig. 4h). Taken together, these results indicate that GA could reduce the expression of RRM2 by inhibiting ERK pathway signaling.

### Gambogic acid and gemcitabine act synergistically against pancreatic cancer cells in vitro by inducing apoptosis

**Fig. 5:**
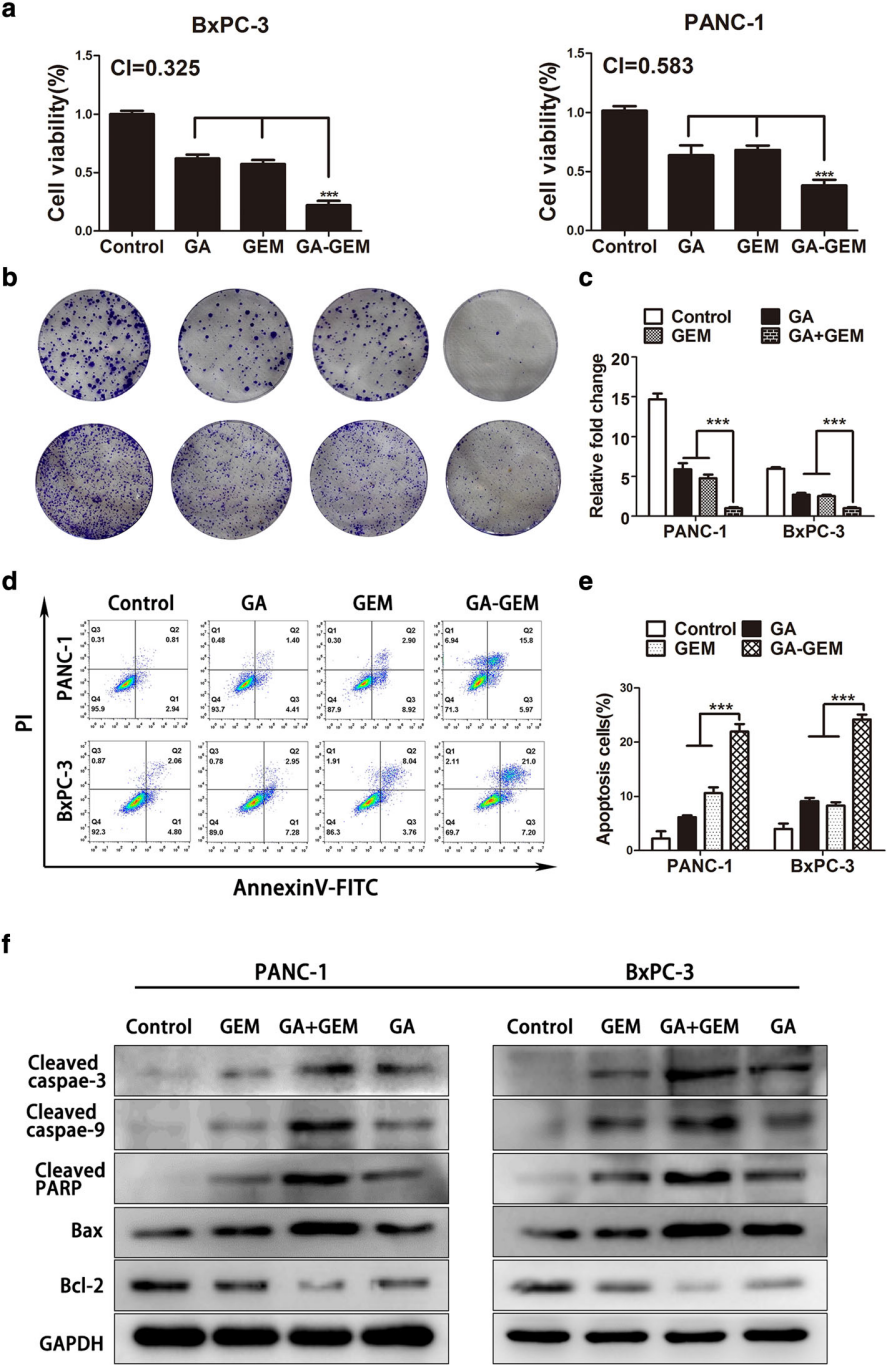
Gambogic acid (GA) sensitized pancreatic cancer cell lines to gemcitabine. In the GA group, PANC-1 and BxPC-3 cells were treated with 1 μM GA for 12 h, and then placed in culture medium for another 48 h. In the GEM group, after incubation with culture medium for 12 h, cells were treated with 5 μM gemcitabine for another 48 h. In the GA-GEM group, cells were pretreated with GA for 12 h, and then treated with gemcitabine for another 48 h. **a** Cell viability was measured using the 4,5-dimethylthiazol-2-yl)-3,5-diphenylformazan (MTT) assay, and the combination index (CI) was calculated using the Chou–Talalay method and CalcuSyn software. **b**, **c** Quantification of the colony formation assay and results. **d**, **e** Apoptosis was detected by flow cytometry. **f** The protein levels of cleaved caspase-3, cleaved caspase-9, cleaved PARP, Bax, and Bcl-2 were detected using western blot analysis. Data are presented as mean ± SD (*n* = 3); *** indicates *P* < 0.001

As RRM2 expression was inhibited by GA, and RRM2 was associated with gemcitabine resistance, we investigated whether GA combined with gemcitabine had a synergistic effect against pancreatic cancer cells. The PANC-1 and BxPC-3 cells were pretreated with GA for 12 h, and then treated with gemcitabine for 48 h. The combined inhibitory effects of GA and gemcitabine on cell growth were found to be superior to those of treatment with GA alone. The combination index was 0.583 for PANC-1 cells and 0.325 for BxPC-3 cells (Fig. 5a). Treatment with GA combined with gemcitabine also significantly inhibited colony formation of PANC-1 and BxPC-3 cells, as compared to treatment with GA or gemcitabine alone (Fig. 5b, c). Flow cytometry analysis following double staining with AnnexinV and PI was performed to detect the ability of GA combined with gemcitabine to induce apoptosis in PANC-1 and BxPC-3 cells. In the combination group, cells were pretreated with GA for 12 h, and then treated with gemcitabine for another 24 h. As illustrated in Fig. 5d and e, the apoptotic rates of PANC-1 and BxPC-3 cells treated with GA combined with gemcitabine were the highest, compared to cells treated with GA or gemcitabine alone. This finding suggests that GA could enhance the ability of gemcitabine to induce apoptosis. To confirm the underlying mechanism, the relevant apoptotic markers, cleaved caspase-3, cleaved caspase-9, cleaved PARP, Bax, and Bcl-2 were determined using western blot analysis. The results showed that the proapoptotic markers, cleaved caspase-3, cleaved caspase-9, and cleaved PARP could be induced by either GA or gemcitabine in PANC-1 and BxPC-3 cells, and could be induced when the cells are subjected to simultaneous treatment with both drugs. In contrast, expression of the anti-apoptotic marker Bcl-2 showed the greatest reduction in the combination treatment group. These data demonstrate that the synergism between gemcitabine and GA could effectively treat pancreatic cancer cells.

### RRM2 reduced GA-induced apoptosis of pancreatic cancer cells

**Fig. 6:**
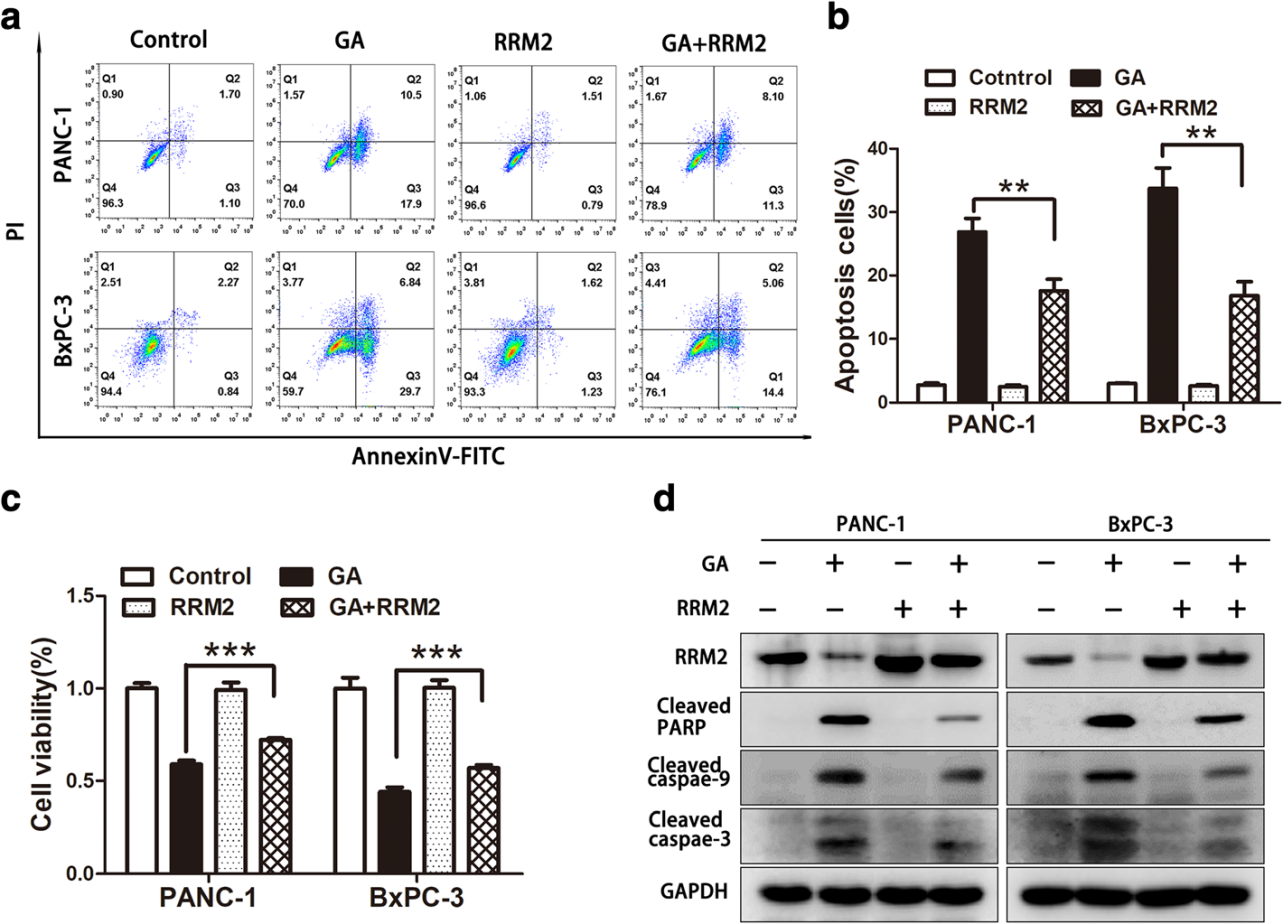
Ribonucleotide reductase subunit-M2 (RRM2) reduced gambogic acid (GA)-induced apoptosis in pancreatic cancer cells. PANC-1 and BxPC-3 cells were transfected with the RRM2 overexpression plasmid over 48 h, and then treated with 2 μM GA for another 24 h. **a**, **b** Apoptotic cells were detected by flow cytometry. **c** Cell viability was detected using the 4,5-dimethylthiazol-2-yl)-3,5-diphenylformazan (MTT) assay. **d** The protein levels of cleaved caspase-3, cleaved caspase-9, cleaved PARP, and RRM2 were detected using western blot analysis. Data are presented as mean ± SD (*n* = 3); ** indicates *P* < 0.01; *** indicates *P* < 0.001

To investigate whether RRM2 affects GA-induced apoptosis of pancreatic cancer cells, we transfected PANC-1 and BxPC-3 cells with the RRM2 overexpression plasmid, following which they were subjected to treatment with GA for another 24 h. Flow cytometry analysis of double stained (with AnnexinV and PI) PANC-1 and BxPC-3 cells was performed to detect apoptosis. The RRM2 overexpression was observed to partially reduce GA-induced apoptosis (Fig. 6a, b), and this was confirmed by the MTT assay (Fig. 6c). The relevant apoptotic markers cleaved caspase-3, cleaved caspase-9, and cleaved PARP were measured using western blot analysis. The results showed that the levels of cleaved caspase-3, cleaved caspase-9, and cleaved PARP induced by GA could be reduced by RRM2 in PANC-1 and BxPC-3 cells. These results suggest that GA could induce apoptosis of pancreatic cancer through the downregulated expression of RRM2.

### Gambogic acid reduces the expression of RRM2 by inhibiting the ERK/E2F1 signaling pathway

**Fig. 7:**
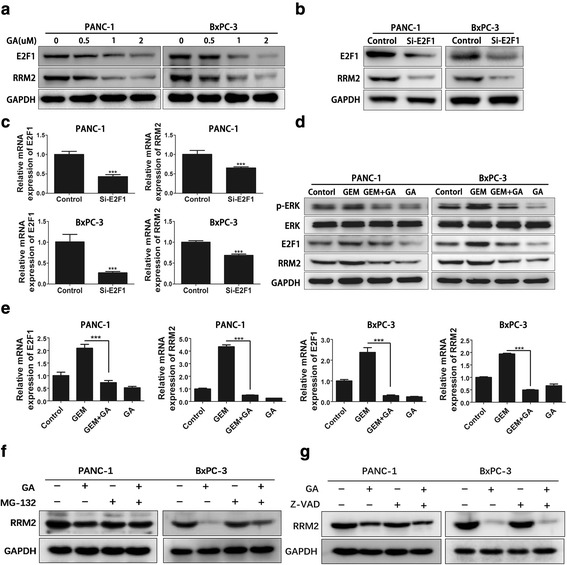
Gambogic acid (GA) reduces the expression of ribonucleotide reductase subunit-M2 (RRM2) by inhibiting the ERK/E2F1 signaling pathway. **a** PANC-1 and BxPC-3 cells were treated with 1, 2, and 4 μM GA for 24 h, respectively. Protein levels of RRM2 and E2F1 were detected using western blot analysis. **b**, **c** PANC-1 and BxPC-3 cells were treated with si-E2F1 for 48 h, and protein levels of RRM2 and E2F1 were detected using western blot analysis (**b**); the mRNA levels of RRM2 and E2F1 were detected by q-PCR (C). (D, E) PANC-1 and BxPC-3 cells were treated with 1 μM GA or 5 μM gemcitabine alone, or in combination (GA with gemcitabine) for 24 h. Expression of the p-ERK, ERK, E2F1 and RRM2 proteins were detected using western blot analysis and q-PCR (**d**). The mRNA expression of E2F1 and RRM2 was detected by q-PCR(**e**). **f** PANC-1 and BxPC-3 cells were pretreated with 2 μM of the proteasome inhibitor, MG-132 for 4 h, and then treated with 2 μM GA for 24 h. Protein expression of RRM2 was detected using western blot analysis. **g** PANC-1 and BxPC-3 cells were pretreated with 20 μM of the pan-caspase inhibitor, Z-VAD-FMK for 4 h, and then treated with 2 μM GA for 24 h. Protein expression of RRM2 was detected using western blot analysis. Data are presented as mean ± SD (*n* = 3); *** indicates *P* < 0.001

One previous study reported that gemcitabine could induce the expression of E2F1 [30]. The transcription factor E2F1 is a promoter that affects the cell cycle and apoptosis, and is a downstream signaling molecule of the ERK signaling pathway [31, 32]. Thus, western blot analysis was performed to prove our hypothesis that the inhibition of RRM2 by GA was due to reduced levels of E2F1 in PANC-1 and BxPC-3 cells. As shown in Fig. 7a, the levels of both E2F1 and RRM2 proteins were reduced in a dose-dependent manner, following treatment with GA for 24 h. When si-E2F1 was used to knock down the expression of E2F1 in PANC-1 and BxPC-3 cells, the protein and mRNA levels of RRM2 were also reduced (Fig. 7b, c), suggesting that our hypothesis was correct. We also found that the ERK signaling pathway was activated and the expression of E2F1 and RRM2 were both elevated when PANC-1 and BxPC-3 cells were treated with gemcitabine for 24 h. Furthermore, GA was able to counteract these elevations in PANC-1 and BxPC-3 cells (Fig. 7d). The mRNA expression of E2F1 and RRM2 was also detected in PANC-1 and BxPC-3 cells treated with GA and gemcitabine using q-PCR (Fig. 7e). A similar trend was observed in protein expression, confirming our previous findings. These results suggest that GA could inhibit the expression of RRM2 by suppressing activation of the MAPK/ERK/E2F1 pathway. Furthermore, we found that the ability of GA to reduce RRM2 expression in PANC-1 and BxPC-3 cells could be impaired by pretreatment with the proteasome inhibitor MG-132 (Fig. 7f), indicating that RRM2 is susceptible to proteasomal degradation. We also investigated whether the caspase pathway would affect the expression of RRM2. Pretreatment with the pan-caspase inhibitor Z-VAD-FMK had no effect on GA-induced downregulation of RRM2 (Fig. 7g).

### Gambogic acid and gemcitabine act synergistically in vivo against pancreatic cancer cells

**Fig. 8:**
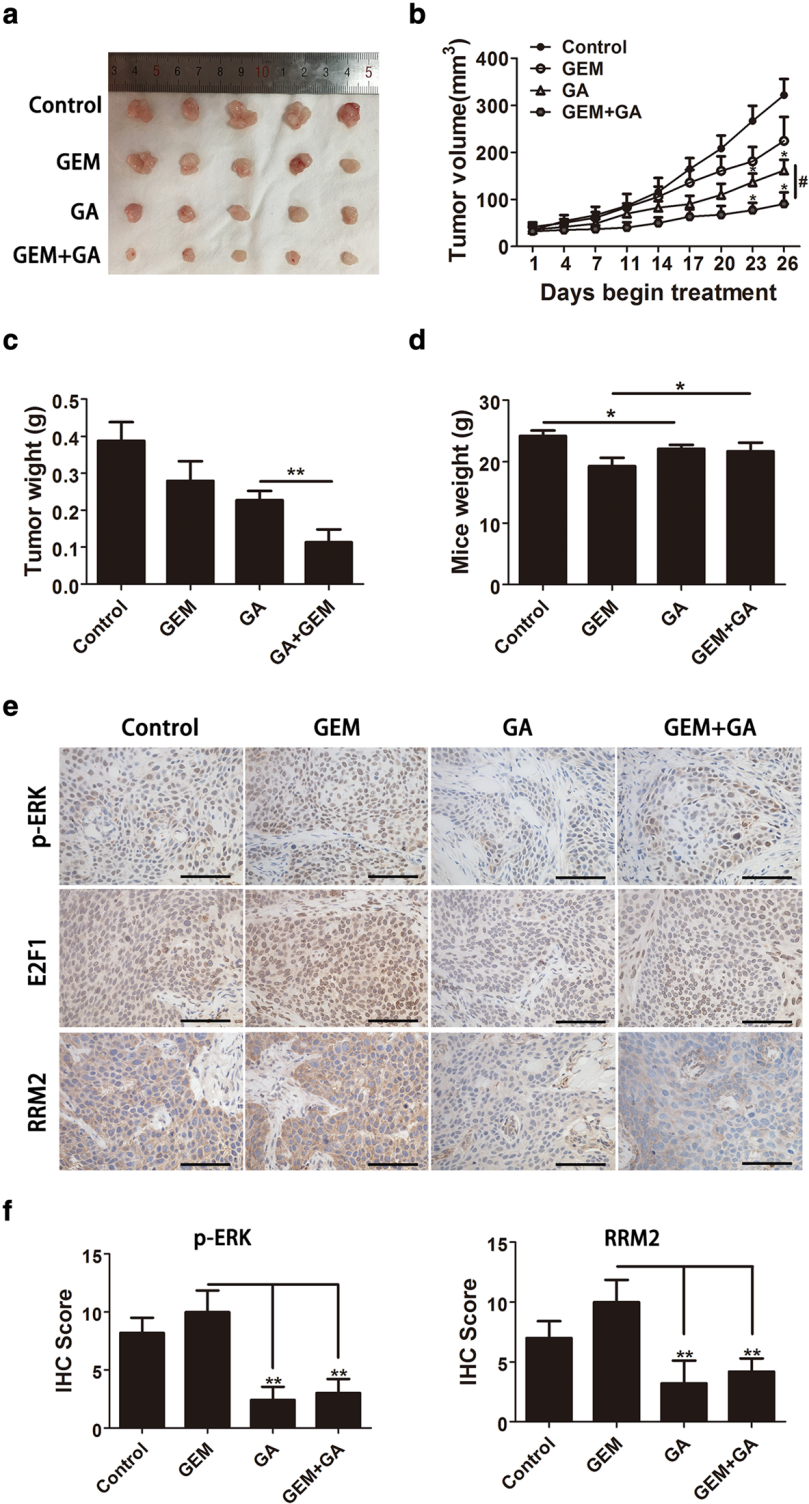
Gambogic acid (GA) and gemcitabine act synergistically against a xenograft tumor model of pancreatic cancer in vivo. Nude mice were used to construct a xenograft tumor model with BxPC-3 cells. **a** Xenograft tumors of four groups. **b** Tumor volume; * indicates the combination group or GA group compared to the control group alone, *P* < 0.05; # indicates the combination group compared to the GA group, *P* < 0.05. **c** Tumor weight. **d** Mice body weight at the end of treatment. **e** IHC staining of p-ERK, E2F1, and ribonucleotide reductase subunit-M2 (RRM2). **f** Quantification of IHC staining of p-ERK and RRM2. Data are presented as mean ± SD (*n* = 5), * indicates *P* < 0.05; ** indicates *P* < 0.01; *** indicates *P* < 0.001

To validate the synergistic effect of GA and gemcitabine against tumor growth in vivo, a xenograft tumor model of pancreatic cancer was produced. The BxPC-3 cell line was used to construct a tumor model in nude mice. Our data showed that GA combined with gemcitabine or used as a single agent reduced tumor growth. The tumor volumes in the treatment group were all reduced compared to those in the control group; however, the degree of inhibition differed. As shown in Fig. 8a and b, the tumor volume in the combination group was significantly smaller than that in the GA and GEM groups, respectively. At 26 days, the mean tumor volume in the control group was 322.03 ± 33.66 mm^3^. In comparison to the control group, the tumor inhibition rates were 49.8%, 30.2%, and 72.9% in the GA, GEM, and combined groups, respectively. Measurements of tumor weight were consistent with those of tumor size (Fig. 8c). These results demonstrate that GA and gemcitabine could act synergistically against pancreatic tumors in vivo. In addition, the body weight of mice in the GA group showed no obvious decline in comparison to the control group, whereas the body weight of mice in the GEM group was significantly lower than that in the control group. The body weight in the combination group was lower than that in the control group, but higher than that in the GEM group (Fig. 8d). These results demonstrate that combined treatment with GA and gemcitabine could effectively, safely, and synergistically inhibit tumor growth in pancreatic cancer.

To determine the mechanism by which GA sensitized pancreatic cancer cells to gemcitabine through inhibition of the ERK/E2F1/RRM2 pathway in vivo, IHC analysis was performed in the tumor tissues of the four experimental groups. The expression of p-ERK, RRM2, and E2F1 was significantly reduced in the combination and GA groups (Fig. 8e), in support of the in vitro results. Quantification of RRM2 and p-ERK staining in tumor tissues was conducted (Fig. 8f).

## Discussion

Pancreatic cancer is highly malignant, and gemcitabine chemotherapy is known to improve survival such cases. Gemcitabine is the first-line treatment choice for pancreatic cancer that induces cell death by inhibiting DNA replication; however, pancreatic cancer is susceptible to gemcitabine resistance [29, 33, 34]. This resistance might be primary resistance or acquired resistance that thereby limits the ability of gemcitabine to improve the prognosis of pancreatic cancer patients [35, 36]. Abnormal gene expression, gene mutation, persistent activation of the signaling pathway, evasion of apoptosis, and tumor stem cells can all lead to resistance of pancreatic cancer cells to chemotherapeutics [37, 38]. In vitro studies have shown that relatively low expression of *hENT1* reduces the intake of gemcitabine by pancreatic cancer cells. Continuous activation of NF-κB and the MAPK/ERK signaling pathway could also cause resistance of pancreatic cancer cells to gemcitabine [29, 39, 40].

The drug resistance genes, *RRM1* and *RRM2*, reduce the pharmacological activity of gemcitabine by affecting its metabolism in pancreatic cancer cells [13, 41]. Previous studies suggest that *RRM1* and *RRM2* could predict the prognosis of patients treated with gemcitabine, as they report that relatively high expression of these two genes in pancreatic cancer is associated with a poor prognosis, and patients who do not benefit from gemcitabine treatment tend to have elevated *RRM1* and *RRM2* expression [42, 43]. In addition, downregulation of *RRM1* or *RRM2* in pancreatic cancer cells could increase their chemosensitivity to gemcitabine [12, 13]. We observed that GA effectively inhibited the expression of *RRM2*, but did not affect the expression of *RRM1*. Moreover, GA could induce apoptosis of pancreatic cancer cells by downregulating the expression of *RRM2*. Furthermore, we found that gemcitabine induced the expression of *RRM2* in pancreatic cancer cells (as previous data has shown), and this effect was inhibited by GA. Wang’s study showed that GA could inhibit the expression of *MDR* in breast cancer [44]. Therefore, we examined the mRNA expression of *MDR*, *MRP*, and other drug resistance genes in pancreatic cancer cells treated with GA. The results showed that GA had no effect on the mRNA expression of these genes (data not shown). In addition, we found that GA could downregulate the expression of some stem cell-related genes in pancreatic cancer cells, such as *ALDH* and *CD44* (data not shown). However, these findings need to be further validated by additional in vitro and in vivo experiments.

The *KRAS* mutation is very common in pancreatic cancer, occurring in approximately 90% of all patients, and it can lead to tumor development and drug resistance [45]. The *KRAS* mutation induces the continuous downstream activation of the RAF/MEK/ERK signaling pathway [46]. Activation of this pathway causes resistance of pancreatic cancer cells to gemcitabine, promotes tumor cell survival, and inhibits apoptosis [36, 47]. Furthermore, Phase III clinical trials have shown that RAS-targeted drugs do not improve the prognosis of patients with advanced pancreatic cancer [48]. The combination of erlotinib and gemcitabine improves survival in some pancreatic cancer patients, but with few benefits, a finding that could be related to drug resistance during the treatment process [49]. Inactivation of the ERK signaling pathway activates the PI3K/AKT signaling pathway in various tumors, and the PI3K/AKT signaling pathway in turn, could promote cell survival, inhibit apoptosis, and induce drug resistance in pancreatic cancer [11, 28]. We found that GA inhibited both the ERK signaling pathway and the AKT signaling pathway. This dual target inhibitory effect might be more potent in inducing chemotherapy resistance. Furthermore, we found that GA could suppress the expression of *RRM2* by inhibiting the ERK signaling pathway. We also found that GA-induced downregulation of *RRM2* could be reduced by inhibition of the proteasome pathway. Previous studies report that the NF-κB signaling pathway could also be inhibited by GA [22]; however, the opposite was observed in oral cancer [50]. Nevertheless, as a traditional Chinese medicine extract, GA exerted a multi-target inhibitory effect, and might have the potential for further therapeutic use in pancreatic cancer.

The transcription factor E2F1 regulates cell proliferation. Furthermore, the ERK signaling pathway can activate E2F1, which is highly expressed in various tumors [30]. However, the role of E2F1 in tumors, such as those of gastric and pancreatic cancers is still debatable. High expression of E2F1 is associated with a poor prognosis, and inhibition of its expression promotes apoptosis [51]. In contrast, high expression of E2F1 promotes apoptosis in colorectal cancer, and is associated with a favorable prognosis; its high expression also induces insensitivity to treatment with 5-fluorouracil (5-FU) in colorectal cancer patients [52]. Studies in pancreatic cancer show that inhibition of E2F1 expression could minimize gemcitabine resistance [30]. The expression of RRM2 could also be regulated by E2F1 via transcription [30]. Our data suggest that GA inhibits the expression of E2F1 and RRM2. In addition, administration of ERK signaling pathway inhibitors could achieve similar effects, which is consistent with the findings of previous studies [31]. When E2F1 was downregulated with si-RNA, we found that the expression of *RRM2* was also downregulated. These results demonstrate that GA could inhibit the expression of E2F1 by inhibiting the activation of the ERK signaling pathway and subsequently suppressed the expression of RRM2.

One of the mechanisms of resistance in pancreatic cancer cells against gemcitabine is apoptosis evasion [38]. Apoptosis is induced by GA through the caspase pathway. Our study found that GA promotes the sensitivity of pancreatic cancer cells to gemcitabine. When pancreatic cancer cells were pretreated with GA for 12 h, and then treated with gemcitabine, apoptosis was evidently stronger than it was with single-drug treatment. Furthermore, the expression of cleaved caspases-3 and 9, cleaved-PARP, and other pro-apoptotic proteins were all increased. These findings illustrate the synergy between GA and gemcitabine. Most remarkably, our in vivo study showed that combined treatment with gemcitabine and GA could show greater efficacy in the inhibition of tumor growth. At the same time, IHC results also demonstrated that GA inhibits phosphorylated ERK and reduces the expression of E2F1 and RRM2. These data suggest that GA could effectively promote the chemosensitivity of gemcitabine in pancreatic cancer. In addition, the body weight of nude mice that were treated with GA and gemcitabine was higher than that of those treated with gemcitabine alone. This finding indicates that GA is relatively safe and could minimize the side effects caused by gemcitabine alone. The Chinese Food and Health Association has approved GA in the second phase of clinical trials for the treatment of lung cancer [53].

The present study also had limitations. Further clinical research is necessary to explore the optimal use, dosage, and treatment schedules of GA in the treatment of pancreatic cancer patients.

## Conclusions

Our study demonstrated that GA increases the efficacy of gemcitabine in pancreatic cancer both in vitro and in vivo, and that the combination of these two drugs could effectively inhibit tumor growth. More notably, our study demonstrated that GA could suppress the expression of RRM2 by inhibiting the ERK / E2F1 signaling pathway. Taken together, these data suggest the potential effectiveness of combination treatment with GA and gemcitabine in patients with pancreatic cancer. However, follow-up clinical studies are necessary to validate the associated effects.

## Additional files

Additional file 1: Figure S1. Pancreatic cancer cell lines PANC-1, BxPC-3, SW1990, and MIA PaCa-2, and the normal pancreatic cell line HPDE were treated with increasing concentrations of gambogic acid (GA) for 24 h; Cell viability was measured using the 4,5-dimethylthiazol-2-yl)-3,5-diphenylformazan (MTT) assay. Data are presented as mean ± SD. (TIFF 431 kb)

Additional file 2: Figure S2. Apoptosis inhibitor Z-VAD-FMK reduced gambogic acid (GA)-induced apoptosis of pancreatic cancer cells. PANC-1 and BxPC-3 cells were pretreated with the pan-caspase inhibitor Z-VAD-FMK (10 μM) for 4 h, and then treated with 2 μM GA for 24 h. (A) Apoptotic cells were detected by flow cytometry. (B) Cell viability was detected using the 4,5-dimethylthiazol-2-yl)-3,5-diphenylformazan (MTT) assay. (C) Protein levels of cleaved caspase-3, cleaved caspase-9, and cleaved PARP, were detected using western blot analysis. Data are presented as mean ± SD (*n* = 3); *** indicates *P* < 0.001. (TIFF 2083 kb)

## Abbreviations

ALDH: Aldehyde dehydrogenase
CI: Combination index
DAB: Diaminobenzidine
DMEM: Dulbecco’s modified Eagle medium
DMSO: Dimethyl sulfoxide
ERK: Extracellular signal-regulated kinase
GA: Gambogic acid
GAPDH: Glyceraldehyde 3-phosphate
GEM: Gemcitabine
HPDE: Human pancreatic ductal epithelial
IHC: Immunohistochemistry
MAPK: Mitogen-activated protein kinase
*MDR*: Multiple drug resistance (gene)
MTT: 4,5-dimethylthiazol-2-yl)-3,5-diphenylformazan
NF-κB: Nuclear factor κB
PBS: Phosphate buffered saline
PCR: Polymerase chain reaction
RIPA: Radioimmunoprecipitation assay
RNR: Ribonucleotide reductase
