# Supplementary figures and images for: Gambogic acid sensitizes gemcitabine efficacy in pancreatic cancer by reducing the expression of ribonucleotide reductase subunit-M2 (RRM2)
Source: J Exp Clin Cancer Res. 2017 Aug 10;36:107. doi: 10.1186/s13046-017-0579-0 (PMC5553806; doi:10.1186/s13046-017-0579-0)

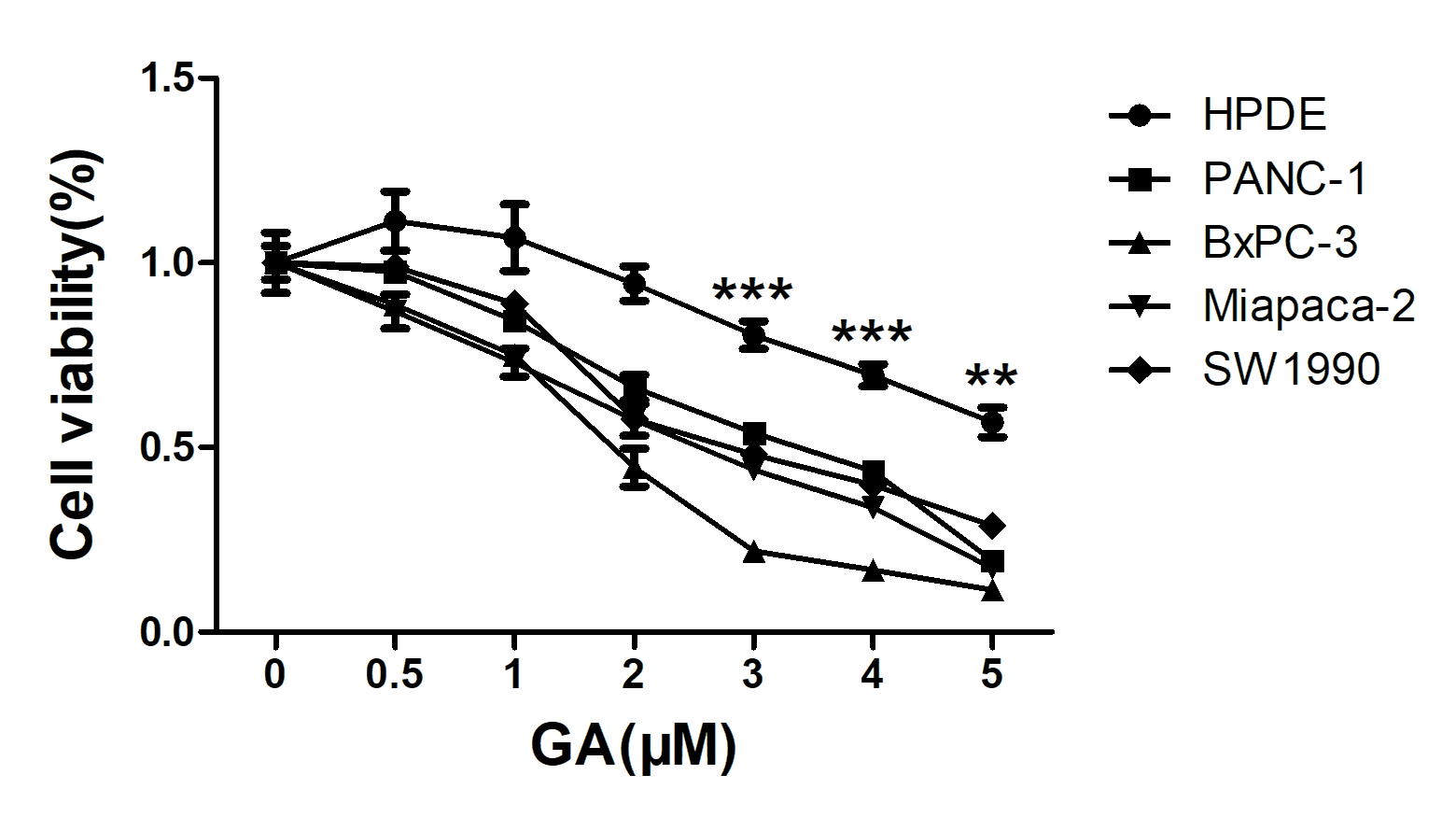

Supplement: Supplementary file 1 — Pancreatic cancer cell lines PANC-1, BxPC-3, SW1990, and MIA PaCa-2, and the normal pancreatic cell line HPDE were treated with increasing concentrations of gambogic acid (GA) for 24 h; Cell viability was measured using the 4,5-dimethylthiazol-2-yl)-3,5-diphenylformazan (MTT) assay. Data are presented as mean ± SD. (TIFF 431 kb) [file 13046_2017_579_MOESM1_ESM.tif]

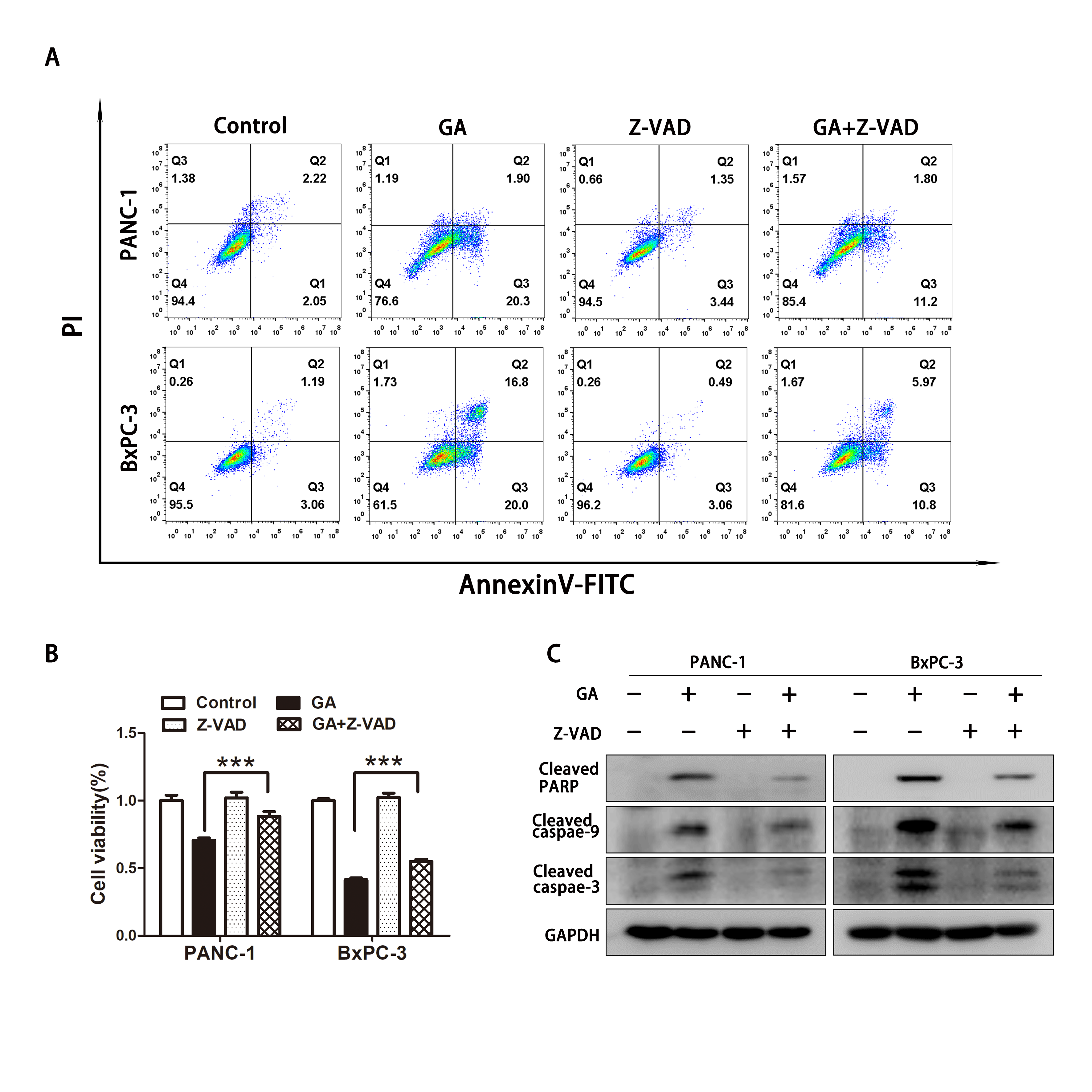

Supplement: Supplementary file 2 — Apoptosis inhibitor Z-VAD-FMK reduced gambogic acid (GA)-induced apoptosis of pancreatic cancer cells. PANC-1 and BxPC-3 cells were pretreated with the pan-caspase inhibitor Z-VAD-FMK (10 μM) for 4 h, and then treated with 2 μM GA for 24 h. (A) Apoptotic cells were detected by flow cytometry. (B) Cell viability was detected using the 4,5-dimethylthiazol-2-yl)-3,5-diphenylformazan (MTT) assay. (C) Protein levels of cleaved caspase-3, cleaved caspase-9, and cleaved PARP, were detected using western blot analysis. Data are presented as mean ± SD (n = 3); *** indicates P < 0.001. (TIFF 2083 kb) [file 13046_2017_579_MOESM2_ESM.tif]
